# Unsupervised machine learning algorithms identify expected haemorrhage relationships but define unexplained coagulation profiles mapping to thrombotic phenotypes in hereditary haemorrhagic telangiectasia

**DOI:** 10.1002/jha2.746

**Published:** 2023-07-03

**Authors:** Ghazel Mukhtar, Claire L. Shovlin

**Affiliations:** ^1^ National Heart and Lung Institute Imperial College London London UK; ^2^ Imperial College School of Medicine London UK; ^3^ Specialist Medicine Imperial College Healthcare NHS Trust London UK; ^4^ NIHR Imperial Biomedical Research Centre London UK

**Keywords:** anaemia, genetic disorders, haemorrhage, high‐dimensional data, t‐SNE

## Abstract

Hereditary haemorrhagic telangiectasia (HHT) can result in challenging anaemia and thrombosis phenotypes. Clinical presentations of HHT vary for relatives with identical casual mutations, suggesting other factors may modify severity. To examine objectively, we developed unsupervised machine learning algorithms to test whether haematological data at presentation could be categorised into sub‐groupings and fitted to known biological factors. With ethical approval, we examined 10 complete blood count (CBC) variables, four iron index variables, four coagulation variables and eight iron/coagulation indices combined from 336 genotyped HHT patients (40% male, 60% female, 86.5% not using iron supplementation) at a single centre. T‐SNE unsupervised, dimension reduction, machine learning algorithms assigned each high‐dimensional datapoint to a location in a two‐dimensional plane. k‐Means clustering algorithms grouped into profiles, enabling visualisation and inter‐profile comparisons of patients’ clinical and genetic features. The unsupervised machine learning algorithms using t‐SNE and k‐Means identified two distinct CBC profiles, two iron profiles, four clotting profiles and three combined profiles. Validating the methodology, profiles for CBC or iron indices fitted expected patterns for haemorrhage. Distinct coagulation profiles displayed no association with age, sex, C‐reactive protein, pulmonary arteriovenous malformations (AVMs), *ENG*/*ACVRL1* genotype or epistaxis severity. The most distinct profiles were from t‐SNE/k‐Means analyses of combined iron‐coagulation indices and mapped to three risk states – for venous thromboembolism in HHT; for ischaemic stroke attributed to paradoxical emboli through pulmonary AVMs in HHT; and for cerebral abscess attributed to odontogenic bacteremias in immunocompetent HHT patients with right‐to‐left shunting through pulmonary AVMs. In conclusion, unsupervised machine learning algorithms categorise HHT haematological indices into distinct, clinically relevant profiles which are independent of age, sex or HHT genotype. Further evaluation may inform prophylaxis and management for HHT patients’ haemorrhagic and thrombotic phenotypes.

## 1 INTRODUCTION

Hereditary haemorrhagic telangiectasia (HHT) is a multisystemic disorder affecting approximately one in 6000 individuals, that displays significant clinical variability [1, 2, 3, 4]. HHT is caused by a single pathogenic DNA variant, usually in the *ENG, AVCRL1* or *SMAD4* genes which are expressed in endothelial cells [5]. The result of these variants is the development of fragile telangiectatic vessels prone to haemorrhage and arteriovenous malformations (AVMs) at characteristic sites [6, 7]. While HHT can be defined clinically by three of four Curaçao Criteria (nosebleeds, telangiectasia, visceral involvement and family history) [8], the converse is not true and possession of a causative HHT pathogenic variant may be associated only with the criterion that precipitated the genetic test [9].

For an individual diagnosed with HHT, it is not possible to predict what the exact consequences will be. Recurrent haemorrhage and iron deficiency anaemia are the most recognised features and are discussed further below. Additionally, extensive evidence accrued in large HHT populations over the last 30 years indicates that approximately one in two patients will have pulmonary AVMs [10] (higher in *ENG* [11]), approximately one in two patients hepatic AVMs [12] (higher in *ACVRL1* [11]), approximately one in 16 patients cerebral AVMs [13] (higher if include other cerebral vascular malformations [13]), with smaller proportions developing AVMs at other sites. For pulmonary AVMs, most patients will have a significant thrombotic complication, such as ischemic stroke/cerebral infarction [14, 15, 16] or cerebral abscess that is associated with venous thromboemboli (VTE) [17, 18] – both result from paradoxical embolism through the right‐to‐left shunts provided by pulmonary AVMs, and management advice is available from respiratory [19], radiological [20] and neurological [15] specialist groupings. For hepatic AVMs, a longitudinal cohort analysis demonstrated mortality and morbidity rates were 1.1 and 3.6 per 100 person‐years, respectively, with development of iron deficiency anaemia a risk factor for high output cardiac failure [21]. For other AVMs in HHT, current data suggest the majority of affected patients will not have a complication, but when these do occur, consequences for the individual can be life changing.

HHT groupings have tended to focus on AVMs, but the most common problems in HHT result from nosebleeds and anaemia which are usually treated in haematological or ENT Services [1, 4, 22, 23]. The majority of HHT patients experience nosebleeds at such frequency and intensity that their iron losses cannot be replaced by dietary iron intake and iron deficiency results [1, 22, 23, 24]. This may be augmented by gastrointestinal blood loss [1, 22, 23, 24]. Anaemia is the best recognised of the many consequences of iron deficiency [25], although the haemorrhage‐adjusted iron requirement (HAIR) [24] indicated anaemia out of proportion to the intake/haemorrhage paradigm, leading to the identification of low‐grade haemolysis as contributing, alongside haemorrhage, to severe anaemia in HHT [26]. Also of relevance to haematologists, in HHT, iron deficiency is associated with increased risk of ischaemic stroke due to pulmonary AVMs [15, 16], increased risk of high output cardiac failure due to hepatic AVMs [21] and increased risk of VTE [27]. Mechanisms supported by evidence in the HHT population include augmented platelet aggregation to 5HT (serotonin) [16, 28] and elevated Factor VIII [27], in addition to wider consequences of iron deficiency [25]. Separately, high transferrin saturation index (T*f*SI) and use of intravenous iron were independent predictors of cerebral abscess for 403 British HHT patients with pulmonary AVMs [17].

These pathophysiological factors do not explain why the clinical presentations of HHT varies so extensively between relatives with identical casual mutations [29, 30]. For bleeding and iron handling, hypothesis‐driven questions provided evidence of subtle differences between HHT *ACVRL1/ENG* and the rare *SMAD4* cases [31], and between groups of HHT patients categorised by the presence of independent non‐HHT DNA variants in genes causing coagulation and platelet disorders [32]. In order to objectively test if there were further patterns relevant to clinical management, we developed unsupervised machine learning algorithms and applied in a novel approach in HHT. Here, we report that the algorithms identified unexpected categories of patients’ haematological indices particularly in relation to iron and coagulation. Known biological factors could not explain the main profile distinctions, providing new areas for mechanistic, clinically relevant studies.

## 2 METHODS

### 2.1 Patient population

Data were examined from a single HHT Reference Centre at Hammersmith Hospital, Imperial College Healthcare NHS Trust London. At this centre, systematic screening haematological, iron and coagulation tests have been standard of care since 1999, enabling the previous hypothesis‐driven questions from the evolving database [24, 26, 27, 31, 32]. With ethical approval, data from patients genotyped by March 2020 were included in the current study if they either had a causal HHT variant identified in *ENG*, *ACVRL1* or *SMAD4* [5], or met a clinical diagnosis of HHT but were negative on genetic testing at the time of analysis. A definite clinical diagnosis required three of four Curaçao Criteria, namely spontaneous recurrent epistaxis, mucocutaneous telangiectasia at characteristic sites, visceral involvement such as gastrointestinal telangiectasia or AVMs in the lungs, liver or brain, and a first degree relative affected by these criteria [8, 9, 23]. Although there were multiple results for each patient due to repeated clinic visits, only first visit results were examined, proving a single dataset per patient. Coagulation data were excluded for patients using anticoagulants due to significant increases in clotting times. Demographic patient details included age, sex, severe epistaxis (severe daily nosebleeds or bleeds resulting in a HAIR exceeding replacement feasibility using oral iron resulting in intravenous iron or blood transfusion dependency) [32] and use of iron supplements (oral or intravenous) [26]. The causal HHT gene was included, but small sample size did not permit inclusion of the *SMAD4* patient group within the statistical testing.

### 2.2 T‐SNE and k‐Means clustering

For t‐SNE and k‐Means analysis (Table 1), haematological indices were grouped into complete blood count (CBC), iron and clotting indices. For each analysis, patients with incomplete data were removed, as this was incompatible with t‐SNE: sample sizes for each set of clinical indices were therefore different. CBC comprised red blood cell count, haemoglobin concentration, haematocrit, mean corpuscular volume, mean corpuscular haemoglobin, mean corpuscular haemoglobin concentration, red cell distribution width (RDW), mean platelet volume, monocyte count and platelet count. Clotting indices comprised platelet count, serum fibrinogen concentration, prothrombin time (PT) and activated partial thromboplastin time (APTT). Iron indices included serum iron, T*f*SI, ferritin and C‐reactive protein (CRP) as a known confounder (marker of inflammation that increases serum ferritin) [27, 37, 38].

**TABLE 1:** Key definitions. T‐SNE and k‐Means clustering was implemented using Python 3.8.13 (Python Software Foundation, Wilmington, USA) and the packages pandas, numpy, matplotlib and sklearn. Data were normalised on a Standard scaler, such that the maximum value for each index was represented using a 1 and the minimum value using a 0 [33]. Euclidean distances were used to calculate the similarity between two high‐dimensional datapoints [33, 34]. Following this, k‐Means clustering was performed on the t‐SNE embeddings [35], using the silhouette score [36] method to determine the most optimal number of clinical profiles. A scatter plot of the t‐SNE embeddings and k‐Means profiles was then plotted for data visualisation.

Term	Definition
High‐dimensional data	Describes data with a large number of features. In the case of clinical profiling, each dimension is represented by a certain clinical parameter.
Unsupervised machine learning	Algorithms analyse patters in uncategorised data
t‐Distributed stochastic neighbourhood embedding (tSNE)	Unsupervised machine learning algorithm which reduces the dimensions of high‐dimensional data down to two. The two dimensions, known as tSNE embeddings, can be plotted along *x* and *y*‐axes to help visualise the data and make sense of patterns. Similar datapoints are represented as closer together in space and dissimilar ones as further apart. t‐SNE unsupervised, dimension reduction, machine learning algorithms therefore assign each high‐dimensional datapoint to a location in a two‐dimensional plane, placing similar datapoints closer together and dissimilar ones further apart.
k‐Means clustering	Clustering algorithm groups similar datapoints together into a cluster with distances between datapoints inversely related to their similarity.

### 2.3 Cluster analyses

Profile characteristics were determined using GraphPad Prism 9.3.1 (GraphPad Software Inc, San Diego, USA). No datasets approximated to a Gaussian distribution. Differences in the distributions were compared using Chi‐squared and Fisher's’ exact tests. For continuous variables, Mann–Whitney tests were used for pairwise comparisons and Kruskal–Wallis tests with post‐hoc Bonferroni correction for multiple comparisons.

## 3 RESULTS

### 3.1 Cohort demographics

The demographic details of all analysis cohorts are displayed in Table 2. There were no significant differences in the age, sex or gene distributions between the cohorts calculated by Chi‐squared (all *p* values > 0.05).

**TABLE 2:** Demographic details of the four cohorts: IQR, interquartile range.

Cohort	*N*	Age, years median (IQR)	Female %	*ENG* *%*	*ACVRL1* *%*	*SMAD4* *%*
Complete blood count	336	49.0 (27.0)	59.8	55.4	27.4	3.6
Iron	218	48.5 (24.5)	61.9	48.6	27.4	4.3
Clotting	309	48.5 (26.0)	62.3	54.4	26.6	3.0
Iron/clotting	196	49.0 (25.0)	61.3	50.5	28.4	1.6

*Note* the remaining patients were ’gene negative’ at time of analysis (none of this cohort had *GDF2* variants).

### 3.2 Homogeneous distribution of CBC indices

As noted in Table 2, the CBC cohort (*n* = 336) comprised 40.2% males and 59.8% females with a median age of 49 (interquartile range [IQR] 27) years. Just over half of the patients had an *ENG* variant, one‐quarter an *ACVRL1* variant and one‐twenty‐eighth had a *SMAD4* variant. In 13.4%, no variant was identified in the main causal HHT genes. 26.8% of patients experienced severe epistaxis, 13.5% received iron supplementation and 79.9% had pulmonary AVMs (all patients are screened by thoracic computerised tomography in a service with a pulmonary AVM referral bias).

**FIGURE 1:**
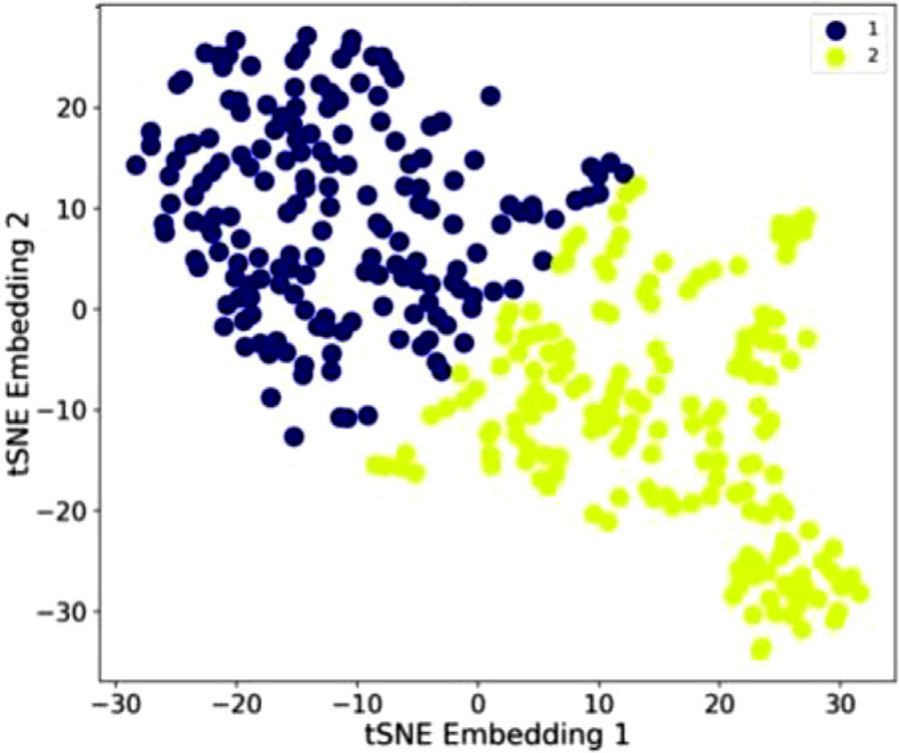
Complete blood count indices. Two‐dimensional scatter plot of the t‐SNE embeddings and k‐Means clusters for CBC indices (*n* = 336). Each datapoint represents a patient. k‐Means clusters are displayed by colour. Distances between datapoints representative of similarity.

The t‐SNE and k‐Means visualisations for CBC profiles are displayed in Figure 1. With distances between datapoints representative of their similarity, CBC profiles appeared homogenous with no distinct separation. All median values were within reference ranges (Table 3). Profile 2 was characterised by significantly lower values for most CBC indices (*p* < 0.0001), except RDW and platelet counts which were significantly higher (*p* < 0.0001).

**TABLE 3:** Details of the CBC indices cohort and two clinical profiles identified: Normal ranges and cohort/profile data for 336 patients with full CBC data. Median (IQR) or % provided. As both sexes were represented, where reference ranges differ by sex, the upper limit provided is the top of the male reference range; lower limit the bottom of the female reference range. MCH, mean corpuscular haemoglobin; MCHC, mean corpuscular haemoglobin concentration. Highlighting indicates values higher (green) or lower (red) than the other profile, and is stronger where significant (***p < 0.0001). Demographic indices for each profile are in non‐ highlighted rows below. Patients included in the profiling had incomplete demographic data, therefore rows may not add up to 100%. Comparisons of CBC and other cohorts are provided in Table 2. * indicates *p* < 0.05.

Measurements	Normal	Cohort (*N* = 336)	Profile (*N* = 174)	Profile 2 (*N* = 162)
Red blood cell count (×10^12^/L)	3.80–5.50	4.73 (0.71)	4.94 (0.69)***	4.55 (0.77)***
Haemoglobin (g/L)	115–170	138 (31)	150 (19.5)***	121 (23)***
Haematocrit (%)	0.36–0.50	0.42 (0.08)	0.45 (0.06)***	0.38 (0.06)***
Mean corpuscular volume (fL)	83–101	88.9 (8.2)	91.1 (5.4)***	84.95 (8.85)***
MCH (pg)	27–32	29.4 (3.2)	30.5 (1.7)***	27.7 (3.1)***
MCHC (g/dL)	31.5–34.5	32.8 (2.3)	33.8 (2.0)***	31.7 (2.6)***
Red cell distribution width (%)	11.5–16.0	13.7 (2.6)	13.0 (1.7)***	15.4 (3.1)***
Mean platelet volume (fL)	9.0–12.1	10.2 (2.2)	10.3 (2.3)	10.1 (1.85)
Monocyte count (×10^9^/L)	0.2–1.0	0.50 (0.3)	0.6 (0.3)	0.5 (0.29)
Platelet count (×10^12^/L)	150–450	263 (0.3)	248 (67.5)***	286 (103.5)***
Age (ys),	–	49 (27)	45 (28)	53.5 (25)*
Sex (% females)	–	59.8	54.0	66.1*
*ENG* genotype (%)	–	55.4	52.9	58.0
*ACVRL1* genotype (%)	–	27.4	27.0	22.8
*SMAD4* genotype (%)	–	3.6	2.3	4.9
Gene not identified (%)	–	13.4	17.8	8.6
Severe epistaxis (%)	–	26.8	16.1	40.0*
Iron supplementation (%)	–	13.5	8.7	18.6*
Pulmonary AVMs (%)	–	79.9	83.1	76.6

Comparing demographics (Table 3), Profile 2 patients were older, with an excess of females, patients with severe epistaxis and patients receiving iron (*p* < 0.05; Figure 1B). Profile 2 patients also had higher RDW and platelet counts consistent with known anaemia associations [37, 38]. There was no difference in HHT genotypes between the profiles.

### 3.3 Two distinct iron indices clusters

**FIGURE 2:**
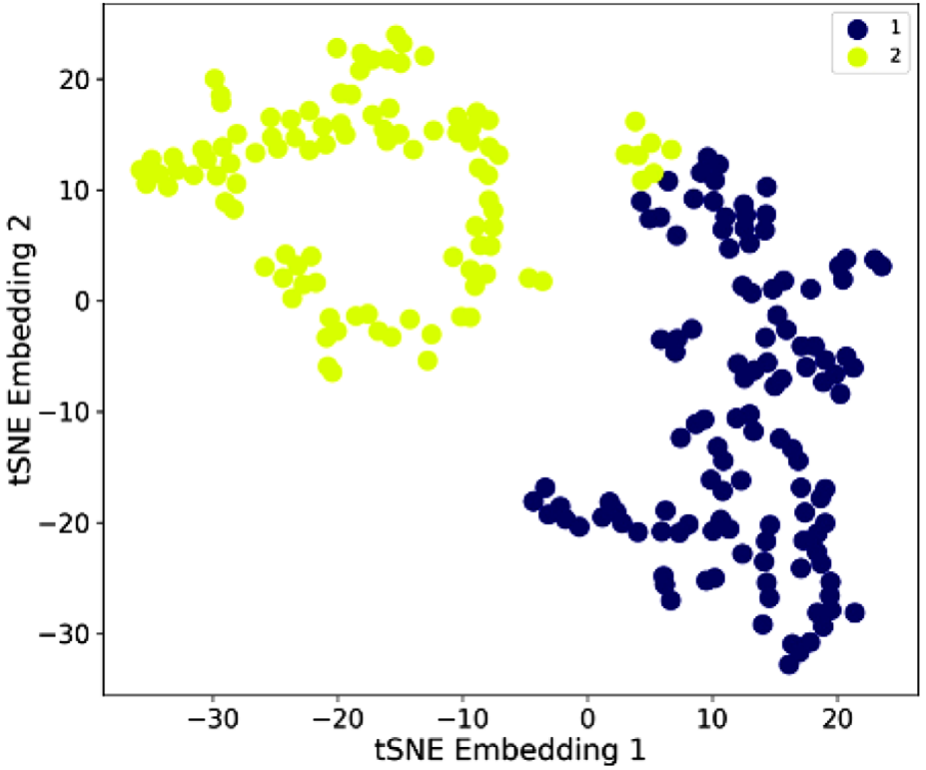
Iron indices. Two‐dimensional scatter plot of the t‐SNE embeddings and k‐Means clusters for CBC indices (*n* = 218). Each datapoint represents a patient. k‐Means clusters are displayed by colour. Distances between datapoints representative of similarity.

Next, iron indices were examined (Figure 2). These separated into distinct regions. Profile 2 patients had lower serum iron, T*f*SI and ferritin and higher CRP (Table 4): Profile 2 iron and T*f*SI median values were below reference ranges, while median ferritin and CRP values were within reference ranges (Table 4). Distinct profiles were evident despite IQRs for iron and T*f*SI similar to median values, and for ferritin, 1.8‐fold greater than the median value.

**TABLE 4:** Details of the iron indices cohort and two clinical profiles identified: Normal ranges and cohort/profile data for 218 patients with full iron indices/CRP data. Median (interquartile range) or % for demographic variables provided. For ferritin, as both sexes were represented within each profile, the lower female and upper male references ranges for ferritin have been combined. Highlighting indicates values significantly higher (green) or lower (red) than the other profile (***p < 0.0001, *p < 0.05). For demographics (non‐highlighted rows below), patients included in the profiling had incomplete demographic data, therefore rows may not add up to 100%. * indicates a difference of *p* < 0.05 between Profiles 1 and 2.

Measurements and demographics	Normal range	Cohort (*N* = 218)	Profile 1 (*N* = 118)	Profile 2 (*N* = 100)
Serum iron (μmol/L)	7–27	13 (11)	18***	6***
Transferrin saturation index (%)	20–40	18 (17.8)	26***	9***
Ferritin (mg/L)	10–150	30.5 (56.3)	44***	18***
C‐reactive protein (mg/L)	<2	1.4 (3.3)	1.0*	1.8*
Age (ys), median (IQR)	–	48.5 (24.5)	51.5 (19.3)	44.5 (27.5)*
Sex (% females)	–	61.9	58.1	66.3
*ENG* genotype (%)	–	48.6	47.0	50.5
*ACVRL1* genotype (%)	–	27.4	33.7	30.3
*SMAD4* genotype (%)	–	4.3	1.00	21.4
Gene not identified (%)	–	18.4	21.4	14.9
Severe epistaxis (%)	–	28.4	16.2	43.3*
Iron supplementation (%)	–	17.9	9.6	21.8
Pulmonary AVMs (%)	–	75.2	79.3	73.3

It had been expected that profiles would relate to haemorrhage severity and/or iron use. Profile 2 patients included a higher proportion experiencing severe epistaxis (12 out of 74 (42%) versus 26 out of 60 (16.2%, *p* < 0.05)), but at presentation, only 18% of the cohort were using iron supplementation and the trend for greater iron use in Profile 2 did not reach significance (Table 4). There was no difference in male/female proportions or genotype, but Profile 2 had a significantly lower median age (Table 4). Profile 2 data were therefore consistent with younger patients with more severe bleeding and lower iron indices.

### 3.4 Four distinct clotting indices clusters

**FIGURE 3:**
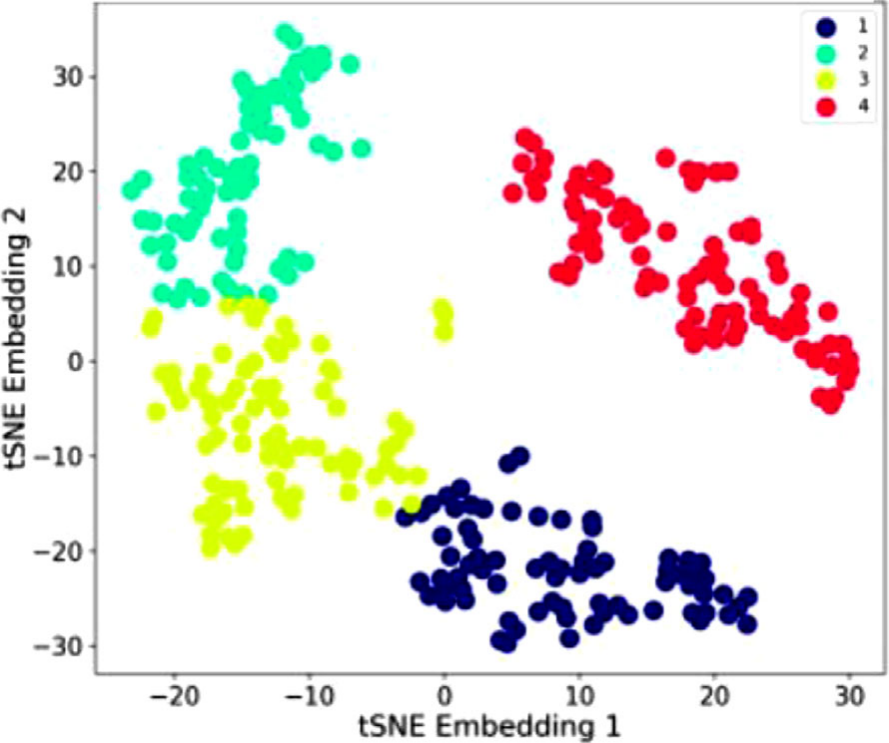
Clotting indices. Two‐dimensional scatter plot of the t‐SNE embeddings and k‐Means clusters for CBC indices (*n* = 309). Each datapoint represents a patient. k‐Means clusters are displayed by colour. Distances between datapoints representative of similarity.

t‐SNE and k‐Means visualisations for clotting indices, yielded four profiles (Figure 3), three (1–3) more distinct to the 4th. As shown in Table 5, platelet counts differed between all profiles (*p* < 0.0001): Profile 1 was characterised by the lowest platelet count, with moderately long PT and APTT. Profile 2 was characterised by the highest median platelet count and shortest PT and APTT; Profile 3 by moderately high platelet count and moderately short PT and APTT; and Profile 4 by moderately low platelet count and significantly longer APTT than the remaining profiles (*p* < 0.0001).

**TABLE 5:** Details of the clotting indices cohort and four clinical profiles identified: Normal ranges and mean indices in 309 patients with full clotting indices data. APTT, activated partial thromboplastin time. Highlighting indicates lowest (red), second lowest (yellow), second highest (orange) and highest (green) values for each clustered parameter. (***p < 0.0001). For demographics (non‐highlighted rows below), patients included in the profiling had incomplete demographic data, therefore rows may not add up to 100%). * indicates a difference of *p* < 0.05 in demographics between profiles.

Measurements and demographics	Normal range	Cohort (*N* = 309)	Profile 1 (*N* = 73)	Profile 2 (*N* = 68)	Profile 3 (*N* = 80)	Profile 4 (*N* = 184)
Platelet count (×10^9^/L)	150–450	261 (87)	213.5 (43.3)***	341 (50)***	269 (25)***	240 (76)***
Fibrinogen (g/L)	1.8–3.6	3.05 (1.0)	2.87 (0.93)	3.14 (0.9)	3.05 (1.05)	3.13 (0.93)
Prothrombin time (s)	9.6–11.6	11.1 (2.5)	11.1 (0.8)	10.5 (0.6)	10.7 (0.7)	14.3 (1.2)
APTT (s)	24–32	26.8 (4.1)	26.0 (2.35)	25.7 (3.3)	25.8 (3.0)	30.0 (3.3)***
Age ys, median (IQR)	–	48.5 (26)	45.5 (23.8)	47 (24)	48 (29)	52 (23.8)
Sex (% females)	–	62.3	62.5	58.2	57.1	72.5
*ENG* genotype (%)	–	54.4	52.5	54.4	48.1	63.8
*ACVRL1* genotype (%)	–	26.6	26.3	27.9	35.1	15.9
*SMAD4* genotype (%)	–	3.0	2.5	5.1	2.6	1.5
Gene not identified (%)	–	15.7	18.7	12.6	14.2	17.4
Severe epistaxis (%)	–	29.3	16.0	35.3	20.7	36.0
Iron supplements (%)	–	13.4	11.3	24.1	3.9	14.5
Pulmonary AVMs (%)	–	78.1	84.3	67.4	72.6	72.6

Across the four profiles there was no difference in median age, male/female proportions or genotype (Table 5). Trends for Profiles 2/4 patients to have more severe epistaxis did not reach significance. There was a difference in the proportion of patients receiving iron supplementation, being highest in Profile 2 (green symbols in Figure 3), lowest in Profile 3 (yellow symbols) and similar in Profiles 1 and 4.

### 3.5 Three distinct clusters defined by iron and coagulation indices

**FIGURE 4:**
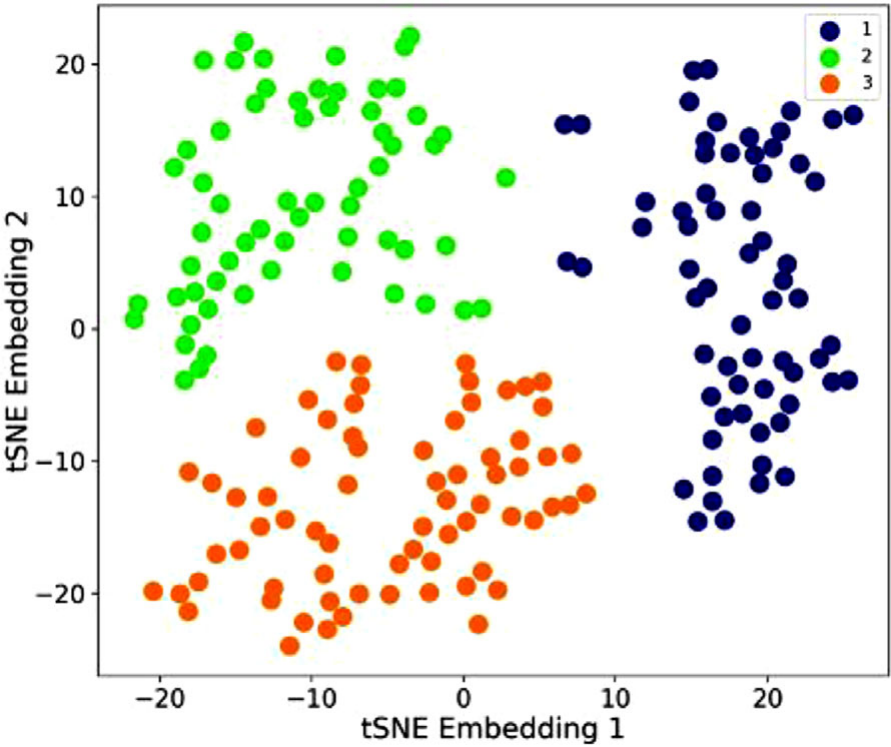
Combined iron and clotting indices. Two‐dimensional scatter plot of the t‐SNE embeddings and k‐Means clusters for iron and clotting indices (*n* = 196). Each datapoint represents a patient. k‐Means clusters are displayed by colour.

Finally, coagulation indices were examined in conjunction with the iron indices that had discriminated the population. This identified three distinct clusters (Figure 4). As detailed further in Table 6, Profile 1 patients (blue symbols in Figure 4) had the lowest iron, T*f*SI and ferritin and highest platelet counts (*p* values < 0.0001). Profile 2 patients (green symbols) were characterised by longest PT and APTT with moderate serum iron and T*f*SI (*p* < 0.0001). Profile 3 patients (orange symbols) were characterised by the highest iron and T*f*SI (*p* values < 0.0001) and lowest fibrinogen concentration (*p* < 0.05). Wide variability in platelet counts and iron indices were noted.

**TABLE 6:** Details of the combined iron/clotting indices cohort, and three clinical profiles identified: Normal ranges and mean iron/clotting indices in 193 patients with full data. APTT, activated partial thromboplastin time. Highlighting indicates lowest (red), intermediate (yellow) and highest (green) values for each clustered parameter. (***p < 0.0001, *p < 0.05). For demographics ( non‐highlighted rows below), patients included in the profiling had incomplete demographic data, therefore rows may not add up to 100%. * indicates a difference of *p* < 0.05 in demographics between profiles.

Measurements and demographics	Normal range	Cohort (*N* = 193)	Profile 1 (*N* = 63)	Profile 2 (*N* = 61)	Profile 3 (*N* = 69)
Serum iron (μmol/L)	7–27	13.0 (11.0)	6.0 (6.3)***	14.0 (15.5)***	18.0 (14.8)***
T*f*SI (%)	20–40	18.0 (18.25)	8.0 (6.3)***	18.5 (15.5)***	27.0 (14.8)***
Ferritin (μg/L)	10–150	31.0 (57.0)	14.0 (25.3)***	45.0 (60.5)	40.0 (51.5)
C‐reactive protein (mg/L)	<2	1.25 (3.13)	1.65 (3.3)	1.00 (3.18)	1.30 (2.43)
Platelet count (×10^9^/L)	150–400	257 (80)	293 (99)***	240 (67)	249.5 (70.3)
Fibrinogen (g/L)	1.8–3.6	3.03 (0.94)	3.05 (1.0)	3.33 (1.1)	2.83 (0.76)*
Prothrombin time (s)	9.6–11.6	11.3 (3.1)	10.65 (0.8)	14.1 (2.6)***	11.0 (2.18)
APTT (s)	24–32	27.0 (4.0)	25.4 (3.0)	29.9 (2.6)***	26.1 (21.8)
Age ys, median (IQR)	–	49.0 (25.0)	45.0 (26.0)	47.0 (28.3)	60.0 (27.0)
Sex (% females)	–	61.3	54.9	59.5	68.8
*ENG* genotype (%)	–	50.5	47.1	46.8	57.8
*ACVRL1* genotype (%)	–	28.4	33.3	26.6	26.6
*SMAD4* genotype (%)	–	1.55	5.88	0	0
Gene not identified (%)	–	19.6	13.7	26.6	15.6
Severe epistaxis (%)	–	30.3	34.7	19.5	37.5
Iron supplements (%)	–	16.0	15.7	13.9	18.8
Pulmonary AVMs (%)	–	73.3	64.3	73.5	77.8

Unlike the earlier profiles, there was no significant difference in the patients’ ages, sex or clinical features for the combined iron and clotting profiles. The Profile 1 combination of lowest iron, T*f*SI and ferritin with highest platelet count is reminiscent of the data that thrombocytosis accompanies acute haemorrhage with iron deficiency anaemia shown to be associated with higher platelet counts [37,38]. Of note, within the combined iron and clotting profiles, all *SMAD4* patients were within the iron‐deficient profile. Profile 3 is also of interest as it was characterised by highest iron and T*f*SI (*p* values < 0.0001) and lowest fibrinogen concentration. There was no significant difference in the distribution of HHT genes, however the small datasets precluded statistical examination of *SMAD4*.

## 4 DISCUSSION

We have shown that unsupervised machine learning algorithms performing objective categorisation of continuous data in HHT patients without prior assumptions, categorise presentation CBC, iron indices and clotting indices into separate clinical profiles. CBC and iron profiles fitted with expected pathophysiological models, but four distinct coagulation profiles and three distinct combined iron‐coagulation profiles could not be explained by current, clinically recognised features, suggesting other drivers of these differences. While bleeding‐iron deficiency considerations are centre stage in HHT, modified coagulation is not despite high rates of VTE [27, 39], and the challenging risk–benefit considerations when using anticoagulation therapy [40, 41, 42, 43).

The main strength of the profiling was the use of unsupervised machine learning algorithms. Previous studies have identified trends by initially categorising the data based on an outcome variable pre‐hypothesised to contribute to clinical variability [5, 14, 16, 17, 21, 24]. This study identified categories without prior assumption of possible determinants. It is widely accepted that reference ranges for several CBC indices are higher in males [38] and t‐SNE and k‐Means’ ability to discriminate sex in CBC profiles validates the methodology. Similarly, more iron‐deficient pictures are expected for patients with more severe haemorrhage [5, 37, 38] and the methodology demarcated an iron indices profile in younger patients with more severe bleeding (Table 4), a group already shown to have poorer long‐term outcomes [44]. Further, by purposefully not excluding specific subgroups other than patients using anticoagulants, this study identified clotting and combined iron‐clotting categorisations that were not associated with any factors routinely used to categorise patients in clinical practice. The study cohorts were proportionately large for a rare genetic disorder, and differences between iron profiles were detected with a power of *α* = 0.05 *β* = 0.99 for iron and T*f*SI, and *β* = 0.84 for ferritin, using the standard deviations of the iron cohort.

Limitations are that the clinical profiles are largely descriptive, and future research should aim to determine the biological drivers of this categorisation. The presence of known comorbidities with the potential to confound clinical indices was not fully explored. However, the sparsity of shared concurrent diagnoses, together with normal electrolytes and renal function [5, 26, 27, 31, 32] testified against overt disease contributions, and there was no difference in CRP between the profiles to suggest acute phase inflammatory responses were contributing. Other limitations pertain to iron indices: serum iron and T*f*SI show diurnal variation, and although variability was partially controlled for due to the routine afternoon timings of blood draws [16, 27], this may have contributed to the large IQRs within the iron indices. Only one in seven patients were using iron (oral or intravenous) at the time of these first clinic datapoints, with final proportions less than 25% in all profiles. Thus, while there are clinical data demonstrating iron treatment responses differ biochemically [45, 46], and clinically in HHT patients [45, 47], these could not be addressed by the current study design. Additionally, the small sample size did not permit for inclusion of the *SMAD4* patient group within the statistical testing, although all *SMAD4* patients were found within the iron‐deficient profile of the combined iron and clotting profiles, supporting the data recently presented by Sharma et al. [31]

CBC and iron indices examined in isolation discriminated patients with more severe bleeding. There was no significant difference in sex distribution for iron indices, which was surprising since male reference ranges for iron indices are typically higher than in females [37, 38]. When profiles incorporated coagulation indices, overlaps between the distinct profiles and recognised pathophysiology were harder to ascertain, although for combined iron and clotting indices, Profile 1 could fit with a ‘simple’ haemorrhage/iron depletion model: As seen in Table 6 it had the lowest iron indices, shortest APTT, highest platelet count and more than twice as many patients reporting severe epistaxis as using iron supplements.

For clotting indices in isolation (platelet count, fibrinogen, APTT and PT), platelet counts differed between all four profiles, though the most dissimilar profile appeared to be driven by a significantly longer APTT. Combining clotting and iron indices, serum iron and T*f*SI primarily discriminated the profiles. The longer APTT distinction remained, now in the profile with intermediate iron/T*f*SI where PT was significantly elevated, and the proportions of severe epistaxis and iron users were lowest. The profile with the highest serum iron indices (medians in normal range despite similar ratios of severe epistaxis and iron use to the ‘haemorrhage/ iron depletion’ Profile 1) also had the lowest fibrinogen count and short APTT. The three distinct combined iron‐clotting profiles (Figure 4) echo observations of secondary phenotypic distinctions determined by logistic regression and ROC curve analyses in the larger HHT cohort from which these genotyped populations were derived: Venous thromboembolism was associated with short APPT and low serum iron [27,39] as seen in the Table 6 Profile 1 [blue] ‘haemorrhage/iron depletion’ profile; cerebral abscess (due to paradoxical microorganism emboli through pulmonary AVMs and blood–brain barrier breach) was associated with VTE and high T*f*SI (compare Table 6 Profile 3 [orange]), while ischaemic stroke risk (considered to be mediated by paradoxical platelet emboli through PAVMs) was higher with lower serum iron and higher serum fibrinogen [16] (compare Table 6 Profile 2 [green]). Thus, while mechanisms underlying distinctions between Profiles 2 and 3 require further evaluation, the machine learning appears to have detected patterns where better appreciation may modify risk assessments for primary and secondary prophylaxis.

Taken together, the data support uncharacterised cellular, endocrine, pharmacological or genetic characteristics that may be operating as modifiers in HHT. Neither the possession of *ENG* nor *ACVRL1* variants was significantly associated with any clinical profile. It is not known whether the profiles will only be relevant to iron and coagulation indices in the setting of an HHT vasculopathy, and replication in non HHT cohorts would be valuable.

In conclusion, in this study unsupervised machine learning t‐SNE and k‐Means algorithms categorised HHT patients’ by haematological indices into distinct clinical profiles. These require further evaluation to inform on iron deficiency treatments and the varying haemorrhagic and thrombotic phenotypes exhibited by HHT patients.

## AUTHOR CONTRIBUTION

G. M. performed the machine learning research and C. L. S. performed the population‐based research. G. M. designed the machine learning study and C. L. S. designed the wider population‐based study. G. M. contributed essential software. G. M. analysed the machine learning data and C. L. S. contributed to data interpretations. G. M. wrote the original draft and C. L. S. edited and finalised the manuscript. Both authors have read and agreed to the published version of the manuscript.

## CONFLICT OF INTEREST STATEMENT

The authors have no conflicts of interest to declare.

## FUNDING INFORMATION

The study received infrastructure support from the National Institute for Health Research Imperial Biomedical Research Centre, London, UK.

## ETHICS STATEMENT

The study was conducted in accordance with the Declaration of Helsinki and approved by the Hammersmith and Queen Charlotte's and Chelsea Research Ethics Committee (LREC 2000/5764).

## CLINICAL TRIAL REGISTRATION

The authors have confirmed clinical trial registration is not needed for this submission.

## PATIENT CONSENT STATEMENT

The authors have confirmed individual patient consent statements are not needed for this submission.

## Data Availability

The data that support the findings of this study are not openly available due to reasons of sensitivity. Categorised data that do not risk breaching anonymity are available from the corresponding author upon reasonable request. Access to primary data can only be granted subject to approval by the project ethics board and under a Data Sharing Agreement.
